# Prevalence and determinants of chronic kidney disease in rural and urban Cameroonians: a cross-sectional study

**DOI:** 10.1186/s12882-015-0111-8

**Published:** 2015-07-30

**Authors:** Francois Folefack Kaze, Diane Taghin Meto, Marie-Patrice Halle, Jeanne Ngogang, Andre-Pascal Kengne

**Affiliations:** Department of Internal Medicine and Specialties, Faculty of Medicine and Biomedical Sciences, University of Yaoundé 1, Yaoundé, Cameroon; Higher Institute of Health Sciences, Bangangté, Cameroon; Department of Internal Medicine and Specialties, Faculty of Medicine and Pharmaceutical Sciences, University of Douala, Douala, Cameroon; South African Medical Research Council & University of Cape Town, Cape Town, South Africa

## Abstract

**Background:**

Chronic kidney disease (CKD) is a global public health problem that disproportionally affects people of African ethnicity. We assessed the prevalence and determinants of CKD and albuminuria in urban and rural adults Cameroonians.

**Methods:**

This was a cross-sectional study of 6-month duration (February to July 2014), conducted in the health district of Dschang (Western Region of Cameroon), using a multistage cluster sampling. All adults diagnosed with albuminuria (≥30 mg/g) and/or decreased estimated glomerular filtration rate (eGFR) (<60 ml/min/1.73 m^2^) were re-examined three months later. Logistic regression models were used to relate baseline characteristics with prevalent CKD.

**Results:**

We included 439 participants with a mean age of 47 ± 16.1 years; with 185 (42.1 %) being men and 119 (27.1 %) being urban dwellers. There was a high prevalence of hypertension (25.5 %), diabetes (9.8 %), smoking (9.3 %), alcohol consumption (59.7 %), longstanding use of herbal medicine (90.9 %) and street medications (87.5 %), and overweight/obesity (53.3 %) which were predominant in rural area. The prevalence of CKD was 13.2 % overall, 14.1 % in rural and 10.9 % in urban participants. Equivalents figures for CKD stages G3-G4 and albuminuria were 2.5 %, 1.6 % and 5.0 %; and 12.1 %, 14.1 % and 6.7 % respectively. Existing hypertension and diabetes were associated with all outcomes. Elevated systolic blood pressure and the presence of hypertension and diabetes were the predictors of albuminuria and CKD while urban residence was associated with CKD stages G3-G4.

**Conclusion:**

The prevalence of CKD and albuminuria was high in this population, predominantly in rural area, and driven mostly by the commonest risk factors.

**Electronic supplementary material:**

The online version of this article (doi:10.1186/s12882-015-0111-8) contains supplementary material, which is available to authorized users.

## Background

Chronic kidney disease (CKD) affects about 1 in 10 adults and accounts for millions of premature deaths worldwide [1, 2]. Studies have revealed that people of African ethnicity are at higher risk of CKD, which is credited to be 3–4 times more frequent in Africans than in developed countries [3]. Many Sub-Saharan Africa (SSA) countries are going through epidemiological transitions and are confronted with the double burden of communicable and non-communicable diseases including CKD [3–5]. However, the few epidemiological studies conducted in SSA have revealed huge disparities in the prevalence of CKD in the adult population, with figures ranging from 1.5 to 38 % depending on methods used to diagnose CKD and population characteristics [6–12]. Furthermore, CKD in SSA tend to affect mostly young adults, with hypertension, chronic glomerulonephritis, diabetes, HIV infection, obesity and herbal medicines use being the main potential contributing factors [3, 5, 7, 8, 10, 11].

High prevalence of CKD have been reported in Central African countries [7, 8, 12]. However, existing studies have been based on non-optimal definition of CKD using a single measurement; which is at variance with the 2012 KDIGO guidelines recommendations of using at least two measurements three months apart [13]. As such, available studies have likely provided inaccurate estimates of the disease burden. In the absence of data on the epidemiology of chronic kidney disease in Cameroon, we undertook this study to establish the prevalence and investigate the determinants of CKD in rural and urban settings in the country.

## Methods

### Study design and setting

**Fig. 1:**
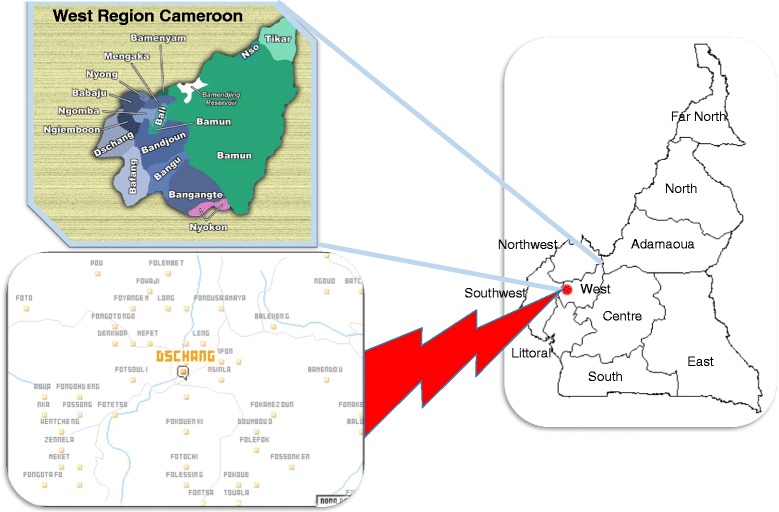
Dschang district in the Western Region of Cameroon

This was a cross-sectional study of 6-month duration (February to July 2014), conducted in the health district Dschang in the Western Region of Cameroon (Fig. 1).

### Study participants

#### Sources of participants

According to the data from the regional delegation of health for the Western Region of Cameroon, Dschang health district is the largest health district in the region, with an estimated population of 309,285 inhabitants in 2012, distributed across 22 health areas (19 rurals and 3 urbans) (2012 annual activity report of the regional delegation of health for Western Cameroon). Dschang is home to the largest university in the Region and therefore has a cosmopolitan population reflecting the ethnic diversity of the country. The adult population in urban areas comprises students, traders, civil servants and middle income earners from private sectors while farmers are predominant in rural area. This study was approved by the Cameroon National Ethics Committee, and all participants provided a written informed consent before enrolment.

#### Eligibility criteria

Eligible participants were adults aged 20 years and above who had been living in the study setting for more than three months. We excluded individuals with serious mental or physical (limb amputation or paralysis) disability, pregnant or breastfeeding women and participants with simultaneous leucocyturia and urine nitrites.

#### Selection of participants

We used a multi-level cluster sampling including the health area (first level), the village (second level), the neighbourhood (third level) and the household (fourth level). The sequence below was followed to select the clusters and corresponding health areas. 1) We first assumed the number of clusters needed to be 30; 2) We then determined the sampling interval (SI) which corresponded to 10310, by dividing the population of Dschang health district by the number of clusters; 3) We determined the first cluster or random number (RN) by selecting the four last numbers of a randomly selected bank note which corresponded to 1399; 4) We next estimated the cluster number size (C_(n)_) from the formula C_(n)_ = RN+(n-1)*SI where n is the cluster number; 5) The various health areas (with their corresponding population size) were then sorted in alphabetic order and progressive cumulative population size estimated (see Additional file 1: Table S1); 6) The last step consisted of selecting the health areas. For a health area to be selected, the size of the corresponding cluster number had to be less than the health area population. This action was repeated until the size of the cluster number became superior to the health area population; we then moved to the following health area in the table. The selected health areas and corresponding clusters are presented in Additional file 1: Table S1.

In a selected health area, one village/neighbourhood was randomly drawn when there was more than one regardless of the population size. The starting point was randomly selected from the market, church, health centre or school in the village/neighbourhood. Thereafter, we randomly selected the direction while the side of road was chosen with the aid of coin toss. We entered consecutively in the households where we randomly selected per household a maximum of two adults aged 20 years and above among those who had been living in the household for more than three months. For each household declining to participate, the next household was selected until the total number required for the cluster size was reached. The cluster size ranged between 14 and 15 subjects. In health areas with many clusters, the corresponding villages and/or neighbourhoods were randomly selected as previously described.

### Variables of interest

The main outcome of interest in this study was CKD defined by the persistence after 3 months of albuminuria (Albumin/Creatinine ratio ≥ 30 mg/g) and/or decreased estimated glomerular filtration rate (eGFR) (<60 ml/min/1.73 m^2^) according to the K/DIGO guidelines [13]. Exposure variables included demographics (age and gender), self-reported existing conditions (hypertension, diabetes and gout), any hypertension (self-reported or screen-detected [i.e. systolic (or diastolic) blood pressure ≥140 (90)], any diabetes (self-reported or screen-detected [fasting capillary glucose ≥126 mg/dl)], lifestyles (alcohol consumption and smoking), use of nephrotoxins [street medications (western drugs, usually of uncertain origins that are sold in shops and regularly along market streets, instead of pharmacies, and without any control) and herbal medicines], overweight or obesity (body mass index [BMI] ≥ 25 kg/m^2^) and blood pressure levels. Potential effect modifier was residency (urban vs. rural).

### Data sources and measurement

Data were collected during household surveys by final year’s undergraduate medical students. Demographics, history of existing conditions, lifestyles and data on the use of nephrotoxins were collected during face-to-face interviews with participants. Blood pressure was measured according to the World Health Organization (WHO) guidelines [14] using an automated sphygmomanometer (OMRON HEM705CP, Omron Matsusaka Co, Matsusaka City, Mie-Ken, Japan) on the right arm with participants in a sitting position after 30 min of rest with a cuff size of 23 x 12 cm or larger for obese individuals. Body weight and height were measured three times and their average used in all analyses. For each participant, 3 ml of whole blood was collected from an antecubital vein for serum creatinine and fasting glycemia (after an overnight fast of at least 8 h), and mid-stream second morning urine collected for dipstick, creatinine and albumin tests. Fasting glycemia and dipstick tests were done immediately after sample collection. The remaining sample was transported in ice to the Biochemistry Laboratory of the Yaounde University Teaching Hospital for further processing. Urine dipstick tests used the CombiScreen 7SL PLUS 7 test strips (Analyticon Biotechnologies AG, D-35104 Lichentenfeis, Germany). Fasting glycemia was performed using One Touch Ultra® easy reader® (LifeScan Europe, Cilag GmbH International, Zug, Switzerland). Serum and urinary creatinine were measured with a kinetic modification of the Jaffé reaction using Human visual spectrophotometer (Human Gesellschaft, Biochemica und Diagnostica mbH, Wiesbaden, Germany) and Beckman creatinine analyzer (Beckman CX systems instruments, Anaheim, CA, USA) while urinary albumin was measured using pyrogallol red-molybdate complex with Teco diagnostics tests (Teco Diagnostics, Anaheim, CA, USA). For any participant with positive dipstick [proteine (≥ trace), blood, leucocytes), albumin/creatinine ratio (ACR) ≥30 mg/g and fasting glycemia of at least 126 mg/dl (for unknown diabetes), another test was performed 2 to 3 weeks after to confirm the results. In participants with estimated glomerular filtration rate (eGFR) < 60 ml/min/1.73 m^2^ according to the MDRD formula and/or urinary albumin/creatinine ratio (ACR) ≥30 mg/g, the chronicity was confirmed on another sample 3 months later.

### Definitions and calculations

Estimated glomerular filtration rate (eGFR, ml/min) corresponded to creatinine clearance. For the main analyses, eGFR was based on the four-variable MDRD (Modification of Diet in Renal Disease) study equation; however, for comparison purpose of the baseline estimates, we also derived the eGFR from the Cockcroft–Gault (CG) formula and the CKD-EPI (Chronic Kidney Disease Epidemiology Collaboration) equations [15–17]. 24-h albuminuria was estimated from Albumin/Creatinine ratio (mg/g). BMI was estimated as weight (kg)/height (m)*height (m).

### Study size

By considering a 10 % prevalence (P) of CKD in adults [1], a precision (I) of 2 %, a correction factor (K) of 2 for the cluster effect, a 95 % confidence interval, the minimal sample size (N) required was 432 subjects using the following formula *N* = [(Zα /2)^2^ PQ/ I^2^] x K.

### Handling of quantitative variables

Age was treated as continuous variable in all analysis while blood pressure, while other quantitative variables were treated both as continuous and categorical variables, based on clinically meaningful stratification. Hypertension was defined as a systolic (SBP) ≥140 mmHg and/or a diastolic blood pressure (DBP) ≥90 mmHg or use of blood pressure lowering medications. Diabetes mellitus was defined as repeated fasting glycemia ≥ 126 mg/dl or use of glucose control agents. A BMI > 25 kg/m^2^ was used to define overweight and obesity. CKD was classified based on GFR and albuminuria categories. GFR categories of CKD included: G1 (eGFR ≥ 90); G2 (eGFR 60–89); G3a (eGFR 45–59); G3b (eGFR 30–44); G4 (eGFR 15–29) and G5 (eGFR < 15). Albuminuria categories of CKD were: A1 (<30 mg/g); A2 (30–300 mg/g) and A3 (>300 mg/g). The following formula was used to convert serum creatinine from Jaffe reaction (SCr_Jaffe_) to standardized serum creatinine (SCr_Standardized_) for use in MDRD and CKD-EPI formulas: SCr_Standardized_ = 0.95*SCr_Jaffe_ – 0.10 [18].

### Statistical analysis

Data analysis used SAS/STAT v9.1 software and the survey analysis procedures (‘*proc surveymeans’, ‘proc surveyreg’ and ‘proc surveylogistic’)* to account for the multilevel sampling design of the study. We have reported the results as means, counts and percentages and the accompanying 95 % confidence intervals. The sampling error was estimated with the use of the Taylor expansion method. Age and sex adjusted logistic regression models were used to investigate the predictors of CKD, CKD stages G3-G4 and albuminuria. A p-value <0.05 was used to indicate statistically significant results. For the main analyses, prevalence and determinants of CKD are based on MDRD derived eGFR. In secondary analyses however, we have also estimated GFR and staged kidney function using the Cockroft-Gault and CKD-EPI equations.

## Results

### Baseline characteristics of the study population

A total of 238 households were included in the survey from which 439 subjects (two participants from 201 households and one participant from the remaining) participated in the study. Eleven (2.5 %) participants from seven (2.9 %) households refused to participate. These households were replaced as described above. The reasons for non-participation were fear (3 participants, 2 households), absence from home after several visits (2 participants, 2 households) and lack of time due to work constraints (6 participants, 3 households).

We included 439 participants aged 47 ± 16.1 years, with 185 (42.1 %) being men and 119 (27.1 %) being urban dweller. In all, 120 (27.3 %) participants had renal abnormalities requiring repeated tests to confirm the chronicity, including 73 (60.8 %) with only albuminuria (ACR ≥ 30 mg/g), 34 (28.3 %) with only decreased eGFR (<60 ml/min/1.73 m^2^) and 13 (10.8 %) with both. The chronicity confirmation test was not performed in 6 (5.3 %) participants including 1 (1.4 %) for albuminuria, 3 (10.3 %) for decreased eGFR and 2 (18.2 %) for both. Reasons for not performing the tests were death (one case) and hospitalisation (5 cases) in other cities for medical care. They were all considered as having negative confirmation tests.

As presented in Table 1, we noticed a high proportion of participants who were unaware of their status for renal disease (80.9 %), hypertension (33 %), diabetes (42.6 %) and gout (56.3 %). There was also a high prevalence of CKD related risk factors in this setting including hypertension (25.5 %), diabetes (9.8 %), smoking (9.3 %), alcohol consumption (59.7 %), longstanding use of herbal medicines (90.9 %) and street medications (87.5 %), and overweight/obesity (53.3 %). This population presented a high prevalence of dipstick proteinuria (19.6 %) and decreased eGFR at 10.7 % based on the MDRD equation, Table 2.

**Table 1:** Baseline clinical characteristics by sex and urban/rural location

Characteristics	Overall	Rural	Urban	p	p sex*residence
n (%)	439 (100)	320 (72.9)	119 (27.1)		
Mean age, years (95 % CI)	47.0 (42.5-51.6)	51.0 (49.1-52.9)	36.5 (32.6-40.3)	<0.0001	0.007
Men, n; % (95 % CI)	185; 42.1 (35.6-48.7)	127; 39.7 (33.0-46.4)	58; 48.7 (37.3-60.2)	0.154	NA
History of renal disease, n; % (95 % CI)				<0.0001	0.640
Yes	1; 0.2 (0.0-0.7)	0	1; 0.8 (0.0-1.7)		
No	83; 18.9 (0.0-38.1)	12; 3.8 (1.9-5.6)	71; 59.7 (38.7-80.6)		
Don’t know	355; 80.9 (61.3-100.0)	308; 96.2 (94.4-98.1)	47; 39.5 (17.8-61.1)		
History of hypertension, n; % (95 % CI)				<0.0001	0.712
Yes	47; 10.7 (8.3-13.1)	33; 10.3 (7.3-13.3)	14; 11.8 (9.7-13.8)		
No	247; 56.3 (46.5-66.1)	148; 46.3 (43.9-48.5)	99; 83.2 (76.6-89.7)		
Don’t know	145; 33.0 (22.4-43.6)	139; 43.4 (40.4-46.4)	6; 5.0 (0.0-11.8)		
History of diabetes, n; % (95 % CI)				<0.001	0.835
Yes	26; 5.9 (2.7-9.1)	23; 7.2 (3.8-10.5)	3; 2.5 (0.0-6.6)		
No	226; 51.5 (38.3-64.7)	122; 38.1 (35.0-41.3)	104; 87.4 (81.2-93.6)		
Don’t know	187; 42.6 (31.1-54.1)	175; 54.7 (51.6-57.8)	12; 10.1 (6.0-14.2)		
History of gout, n; % (95 % CI)				<0.001	0.063
Yes	5; 1.1 (0.6-1.7)	4; 1.3 (0.6-1.9)	1; 0.8 (0.0-1.7)		
No	187; 42.6 (26.6-58.6)	84; 26.3 (22.8-29.7)	103; 86.6 (80.5-92.6)		
Don’t know	247; 56.3 (40.2-72.3)	232; 72.5 (69.0-76.0)	15; 12.6 (6.2-19.0)		
Tobacco use currently or formerly, n; % (95 % CI)	41; 9.3 (7.5-11.2)	34; 10.6 (8.5-12.7)	7; 5.9 (4.5-7.3)	<0.0001	0.009
Alcohol use currently or formerly, n; % (95 % CI)	262; 59.7 (51.3-68.1)	211; 65.9 (60.7-71.1)	51; 42.9 (27.6-58.1)	0.003	0.001
Longstanding use of herbal medicine, n; % (95 % CI)	399; 90.9 (83.8-97.9)	312; 97.5 (96.1-98.9)	87; 73.1 (65.6-80.6)	<0.0001	0.090
Longstanding use of street medications, n; % (95 % CI)	384; 87.5 (77.3-97.7)	306; 95.6 (93.9-97.4)	78; 65.5 (51.6-79.4)	<0.0001	0.064
Mean systolic blood pressure, mmHg (95 % CI)	119.5 (117.0-122.1)	121.2 (119.5-122.9)	115.0 (111.6-118.5)	0.015	0.163
Mean diastolic blood pressure, mmHg (95 % CI)	77.9 (77.0-78.9)	78.1 (77.0-79.3)	77.4 (76.0-78.8)	0.543	0.253
Any hypertension, n (%)	112; 25.5 (22.3-28.7)	92; 28.8 (25.7-31.8)	20; 16.8 (13.0-20.6)	<0.0001	0.077
Mean body mass index, kg/m^2^ (95 % CI)	26.0 (25.7-26.4)	26.0 (25.6-26.4)	26.2 (25.5-26.8)	0.711	0.004
BMI ≥ 25, n; % (95 % CI)	234; 53.3 (49.5-57.1)	169; 52.8 (49.5-56.1)	65; 54.6 (45.6-63.6)	0.696	0.102
Mean waist girth, cm (95 % CI)	88.4 (87.2-89.7)	89.2 (88.0-90.4)	86.3 (84.8-87.9)	0.021	0.277
Mean fasting glycemia, g/l (95 % CI)	0.92 (0.88-0.96)	0.93 (0.88-0.97)	0.91 (0.87-0.95)	0.539	0.387
Any diabetes, n; % (95 % CI)	43; 9.8 (6.6-13.0)	34; 10.6 (7.2-14.0)	9; 7.6 (2.1-13.1)	0.370	0.976

*95 % CI* 95 % confidence intervals

**Table 2:** Baseline kidney function test and urine profile by location

Characteristics	Overall	Rural	Urban	p	p sex*residence
n (%)	439 (100)	320 (72.9)	119 (27.1)		
Dipstick abnormalities, n; % (95 % CI)					
Proteinuria	86; 19.6 (13.5-25.6)	76; 23.8 (18.5-29.0)	10; 8.4 (3.3-13.5)	0.0005	0.155
Leucocyturia	22; 5.0 (2.4-7.6)	10; 3.1 (1.6-4.7)	12; 10.1 (0.0-20.1)	0.034	0.807
Hematuria	2; 0.5 (0.0-1.1)	0	2; 1.7 (0.2-3.2)	<0.0001	0.984
Albuminuria, mg/g, n; % (95 % CI)				0.0004	0.148
<30	353; 80.4 (74.3-86.5)	244; 76.3 (71.0-81.5)	109; 91.6 (86.5-96.7)		
30-300	70; 15.9 (10.0-21.8)	64; 20.0 (15.6-24.3)	6; 5.0 (0.0-11.1)		
>300	16; 3.6 (2.1-5.2)	12; 3.8 (1.9-5.6)	4; 3.4 (1.1-5.6)		
Mean serum creatinine, mg/l (95 % CI)	10.6 (10.0-11.2)	10.3; 4.9 (9.6-10.9)	11.6; 3.9 (10.5-12.8)	0.065	0.037
Mean eGFR, ml/min (95 % CI)					
MDRD	109.5 (104.0-115.0)	110.9; 38.2 (105.2-116.5)	105.8; 48.1 (91.7-119.8)	0.522	0.0005
CKD-EPI	106.4 (101.3-111.5)	107.2; 30.1 (102.6-111.8)	104.2; 38.6 (90.1-118.3)	0.622	0.0003
CG	89.1 (83.2-95.0)	88.2; 35.5 (83.1-93.2)	91.8; 35.4 (78.4-105.1)	0.691	0.0002
Stages of kidney function by eGFR, n; % (95 % CI)					
MDRD (ml/min/1.73 m^2^) >90	303; 69.0 (63.3-74.7)	238; 74.4 (70.4-78.3)	65; 54.6 (38.3-70.9)	0.2681	0.711
60-90	89; 20.3 (15.0-25.5)	53; 16.6 (13.1-20.0)	36; 30.3 (16.7-43.8)		
<60	47; 10.7 (5.4-16.0)	29; 9.1 (3.0-15.1)	18; 15.1 (4.5-25.8)		
CG (ml/min) >90	197; 44.9 (39.7-50.1)	142; 44.4 (40.6-48.2)	55; 46.2 (32.2-60.2)	0.757	0.090
60-90	146; 33.3 (30.6-35.9)	106; 33.1 (29.9-36.3)	40; 33.6 (29.5-37.7)		
<60	96; 21.9 (16.3-27.4)	72; 22.5 (18.2-26.8)	24; 20.2 (5.7-34.6)		
CKD-EPI (ml/min/1.73 m^2^) >90	309; 70.4 (64.7-76.0)	243; 75.9 (72.3-79.6)	66; 55.5 (38.4-72.5)	0.242	0.543
60-90	82; 18.7 (13.6-23.7)	48; 15.0 (11.9-18.1)	34; 28.6 (15.2-41.9)		
<60	48; 10.9 (5.4-16.4)	29; 9.1 (3.0-15.1)	19; 16.0 (3.9-28.0)		
Albuminuria (≥30) and/or eGFR (<60) MDRD	120; 27.3 (20.6-34.5)	96; 30.0 (24.3-35.7)	24; 20.2 (8.3-32.0)	0.162	<0.0001
Albuminuria (≥30) and/or eGFR (<60) CG	157; 35.8 (27.3-44.2)	129; 40.3 (34.5-46.1)	28; 23.5 (7.8-39.3)	0.072	0.022
Albuminuria(≥30) and/or eGFR(<60)CKD-EPI	121; 27.6 (20.6-34.5)	96; 30.0 (24.3-35.7)	25; 21.0 (7.5-34.6)	0.252	<0.0001

*CG* Cockroft-Gault, *CKD-EPI* Chronic kidney disease epidemiology collaboration, *eGFR* Estimated glomerular filtration rate, *MDRD* Modification of Diet in Renal Disease, *95 % CI* 95 % confidence intervals

Compared with their urban counterparts, rural participants were older (51.0 vs. 36.5 years, *p* < 0.001) and less aware of their status for renal disease, hypertension, diabetes and gout (all *p* < 0.001), Table 1. Furthermore, they included higher proportion of alcohol drinkers, longstanding users of street and herbal medicine, and had elevated SBP and waist girth, and higher prevalence of hypertension and albuminuria (all *p* ≤ 0.021), Tables 1 and 2. The prevalence of combined albuminuria and/or decreased MDRD-based eGFR was 30 % (95 % CI: 24.3-46.7) in rural and 20.2 % (8.3-32.0) in urban areas (p = 0.162), Table 2. There were suggestions that urban vs. rural variations in the levels of some characteristics occurred in differential ways between men and women. This was the case for age, tobacco use, alcohol use, body mass index, eGFR and prevalent CKD (all *p* < 0.037 for sex*residence interactions), Tables 1 and 2.

### Prevalence and correlates of albuminuria and chronic kidney disease

As presented in Table 3, the prevalence of albuminuria was 12.1 % (*n* = 53) overall, 14.1 % (*n* = 45) in rural and 6.7 % (*n* = 8) in urban participants (*p* = 0.047). Compared with their non-albuminuric counterparts, participants with albuminuria had higher prevalence of existing hypertension (*p* < 0.0001), diabetes (*p* < 0.003), gout (*p* = 0.0002) and longstanding use of herbal medicine (*p* = 0.0001), higher SBP (p = 0.019), and higher prevalence of any hypertension (*p* = 0.0001) and diabetes (*p* = 0.008). The prevalence of CKD was 13.2 % with 2.5 % at stages G3-G4, Table 3. Equivalents figures were 14.1 % and 1.6 % in rural, and 10.9 % and 5.0 % in urban participants. CKD was associated with history of hypertension (*p* = 0.001), diabetes (*p* = 0.017) and gout (*p* = 0.009), herbal medicines (*p* < 0.0001) and street medications (*p* = 0.007) use, any diabetes (*p* = 0.017) and hypertension (*p* < 0.0001), while stages G3-G4 of CKD were associated with female sex (*p* < 0.0001), tobacco use (*p* < 0.0001), and herbal medicines (*p* < 0.0001) and street medications (*p* < 0.0001) use, Table 3. In males vs. female comparisons, the prevalence of albuminuria was 10.3 % and 13.4 % respectively. Equivalents figures were 10.3 % and 15.3 % for CKD, and 0 % and 4.3 % for stages G3-G4 of CKD.

**Table 3:** Characteristics according to the presence/absence of chronic kidney disease with glomerular filtration rate and albuminuria categories

Variables	CKD			GFR based CKD stages			Albuminuria based CKD stages		
	No	Yes	p	G1-G2	G3-G4	p	A1	A2-A3	p
n (%)	381 (86.8)	58 (13.2)		428 (97.5)	11 (2.5)		386 (87.9)	53 (12.1)	
Sex (Men), n; % (95 % CI)	166; 43.6 (36.7-50.5)	19; 32.8 (20.5-45.0)	0.094	185; 43.2 (36.9-49.5)	0	<0.0001	166 (36.2-49.8)	19; 35.8 (23.7-48.0)	0.230
Rural residence, n; % (95 % CI)	275; 72.2 (47.8-96.6)	45; 77.6 (56.7-98.5)	0.432	315; 73.6 (50.2-97.0)	5; 45.5 (0.0-92.3)	0.077	275; 71.2 (46.5-96.0)	45; 84.9 (70.81-99.0)	0.047
Mean age, years (95 % CI)	46.5 (41.5-51.5)	50.2 (46.7-53.8)	0.191	46.9 (42.4-51.5)	50.6 (40.9-60.3)	0.413	46.5 (41.5-51.5)	50.7 (46.8-54.5)	0.193
History of hypertension, n; % (95 % CI)			0.001			0.766			<0.0001
Yes	34; 8.9 (6.1-11.7)	13; 22.4 (12.7-32.1)		42; 9.8 (7.4-12.2)	5; 45.5 (16.6-74.3)		35; 9.1 (6.4-11.7)	12; 22.6 (12.2-33.1)	
No	226; 59.3 (49.7-69.0)	21; 36.2 (26.3-46.1)		242; 56.5 (47.0-66.1)	5; 45.5 (17.7-73.2)		230; 59.6 (49.6-69.5)	17; 32.1 (24.0-40.1)	
Don’t know	121; 31.8 (21.1-42.4)	24; 41.4 (27.1-55.6)		144; 33.6 (23.0-44.3)	1; 9.1 (0.0-28.2)		121; 31.3 (20.6-42.1)	24; 45.3 (31.4-59.1)	
History of diabetes, n; % (95 % CI)			0.017			0.662			0.003
Yes	16; 4.2 (0.9-7.5)	10; 17.2 (6.5-27.9)		23; 5.4 (2.4-8.4)	3; 27.3 (0.0-60.1)		16; 4.1 (0.8-7.5)	10; 18.9 (8.0-29.7)	
No	206; 54.1 (40.8-67.3)	20; 48.3 (19.1-49.8)		219; 51.2 (38.2-64.1)	7; 63.6 (28.4-98.8)		211; 54.7 (41.1-68.2)	15; 28.3 (17.1-39.5)	
Don’t know	159; 41.7 (29.6-53.9)	28; 48.3 (34.6-61.9)		186; 43.5 (31.9-55.0)	1; 9.1 (0.0-28.2)		159; 41.2 (28.8-53.6)	28; 52.8 (40.1-65.6)	
History of gout, n; % (95 % CI)			0.009			0.566			0.0002
Yes	4; 1.0 (0.4-1.7)	1; 1.7 (1.0-2.4)		5; 1.2 (0.6-1.7)	0		4; 1.0 (0.4-1.7)	1; 1.9 (1.2-2.6)	
No	169; 44.4 (28.2-60.5)	18; 31.0 (14.9-47.2)		181; 42.3 (26.6-58.0)	6; 54.5 (7.7-100)		174; 45.1 (28.5-61.6)	13; 24.5 (12.9-36.2)	
Don’t know	208; 54.6 (38.2-71.0)	39; 67.2 (51.4-83.1)		242; 56.5 (40.7-72.3)	5; 45.5 (0.0-92.3)		208; 53.9 (37.2-70.6)	39; 73.6 (62.1-85.1)	
Tobacco use, n; % (95 % CI)	38; 10.0 (8.0-12.0)	3; 5.2 (0.8-9.5)	0.071	41; 9.6 (7.7-11.5)	0	<0.0001	38; 9.8 (7.8-11.9)	3; 5.7 (1.0-10.3)	0.129
Alcohol use, n; % (95 % CI)	231; 60.6 (51.5-69.8)	31; 53.4 (39.5-67.4)	0.310	259; 60.5 (51.9-69.1)	3; 27.3 (0.0-57.0)	0.068	234; 60.6 (51.3-70.0)	28; 52.8 (38.6-67.1)	0.300
Longstanding use of herbal medicine, n; % (95 % CI)	341; 89.5 (81.5-97.5)	58; 100 (100–100)	<0.0001	388; 90.7 (83.4-97.9)	11; 100 (100–100)	<0.0001	346; 89.6 (81.8-97.4)	53; 100 (100–100)	<0.0001
Longstanding use of street medications, n; % (95 % CI)	330; 86.6 (81.5-97.5)	54; 93.1 (85.7-100.0)	0.007	373; 87.1 (76.7-97.6)	11; 100 (100–100)	<0.0001	335; 86.8 (76.4-97.2)	49; 92.5 (84.1-100.0)	0.025
Mean systolic blood pressure, mmHg (95 % CI)	118.6 (116.6-120.6)	125.6 (118.3-133.0)	0.053	119.3 (117.1-121.5)	128.3 (103.0-153.5)	0.457	118.5 (116.2-120.7)	127.2 (120.7-133.7)	0.019
Mean diastolic blood pressure, mmHg (95 % CI)	77.7 (76.8-78.5)	79.8 (76.0-83.5)	0.294	77.9 (76.8-78.9)	81.5 (70.4-92.6)	0.511	77.6 (76.8-78.4)	80.3 (76.8-83.8)	0.164
Any hypertension, n; % (95 % CI)	89; 23.4 (19.7-27.0)	23; 39.7 (31.9-47.4)	<0.0001	107; 25.0 (21.7-28.3)	5; 45.5 (16.6-74.3)	0.107	90; 23.3 (19.6-27.0)	22; 41.5 (32.7-50.3)	<0.0001
Body mass index ≥ 25, n; % (95 % CI)	200; 52.5 (48.7-56.3)	34; 58.6 (48.1-69.2)	0.196	227; 53.0 (48.8-57.3)	7; 63.6 (39.8-100)	0.526	204; 52.8 (49.3-56.4)	30; 56.6 (46.2-67.0)	0.374
Mean fasting glycemia, g/l (SD)	0.91 (0.87-0.94)	1.02 (0.86-1.18)	0.167	0.91 (0.88-0.95)	1.25 (0.73-1.76)	0.200	0.91 (0.87-0.94)	1.02 (0.85-1.20)	0.191
Any diabetes, n; % (95 % CI)	32; 8.4 (4.7-12.1)	11; 19.0 (8.9-29.0)	0.017	40; 9.3 (6.2-12.5)	3; 27.3 (0.0-0.60)	0.102	32; 8.3 (4.6-12.0)	11; 20.8 (10.3-31.2)	0.008

*CKD* Chronic kidney disease, *GFR* glomerular filtration rate (GFR); *95 % CI* 95 % confidence intervals

### Predictors of albuminuria and chronic kidney disease in age, sex and residency adjusted logistic regressions

Existing hypertension and diabetes were consistently and positively associated with higher risk of all outcomes with however, borderline association between hypertension and stages G3-G4 CKD (*p* = 0.056), Table 4. Elevated SBP and the presence of hypertension and diabetes were the predictors of albuminuria and CKD. Rural residence was negatively and consistently associated with the presence of stages G3-G4 CKD while age and sex did not affect any of the outcomes, Table 4.

**Table 4:** Predictors of chronic kidney disease and albuminuria in age, sex and residence adjusted logistic regressions

Variables	CKD		CKD Stages G3-G4		Albuminuria	
	OR (95 % CI)	*p*	OR (95 % CI)	*p*	OR (95 % CI)	*p*
Sex (men)	0.63 (0.36-1.08)	0.095	<0.001 (<0.001- < 0.001)	<0.0001	0.76 (0.46-1.25)	0.283
Residence (rural)	1.06 (0.52-2.16)	0.880	0.16 (0.04-0.69)	0.008	1.92 (1.06-3.48)	0.031
Age, per years	1.01 (0.99-1.03)	0.178	1.03 (1.00-1.07)	0.066	1.01 (0.99-1.03)	0.293
History of hypertension		<0.0001		0.056		<0.0001
No	1 (reference)		1 (reference)		1 (reference)	
Yes	3.95 (2.09-7.46)		4.74 (0.84-26.58)		4.67 (2.63-8.29)	
Don’t know	2.16 (1.29-3.63)		0.50 (0.03-8.66)		2.38 (1.40-4.03)	
History of diabetes		<0.0001		0.004		<0.0001
No	1 (reference)		1 (reference)		1 (reference)	
Yes	6.64 (2.63-16.75)		4.49 (1.55-13.03)		8.13 (3.17-20.84)	
Don’t know	1.89 (1.09-3.26)		0.17 (0.01-3.11)		2.25 (1.26-4.01)	
History of gout		0.131		<0.0001		0.064
No	1 (reference)		1 (reference)		1 (reference)	
Yes	2.58 (0.96-6.94)		<0.001 (<0.001- < 0.001)		3.13 (1.14-8.59)	
Don’t know	1.74 (0.94-3.23)		1.38 (0.32-5.94)		2.12 (1.04-4.31)	
Tobacco use	0.50 (0.24-1.05)	0.066	<0.001 (<0.001- < 0.001)	<0.0001	0.52 (0.25-1.07)	0.077
Alcohol use	0.75 (0.40-1.39)	0.362	0.32 (0.07-1.41)	0.133	0.65 (0.35-1.19)	0.160
Longstanding use of street medications	1.77 (0.87-3.60)	0.113	Few events		1.17 (0.52-2.63)	0.713
Systolic blood pressure, mmHg	1.01 (1.00-1.02)	0.018	1.02 (0.99-1.05)	0.158	1.01 (1.00-1.02)	0.002
Diastolic blood pressure, mmHg	1.01 (0.99-1.03)	0.175	1.02 (0.97-1.08)	0.342	1.01 (1.00-1.03)	0.038
Any hypertension	2.07 (1.38-3.11)	0.0004	2.85 (0.65-12.45)	0.163	2.19 (1.47-3.25)	0.0001
Body mass index ≥ 25 kg/m^2^	1.34 (0.89-2.02)	0.157	1.55 (0.35-6.83)	0.564	1.22 (0.84-1.78)	0.288
Any diabetes	2.43 (1.15-5.16)	0.021	3.88 (0.75-20.19)	0.107	2.74 (1.26-5.98)	0.011

*CKD* Chronic kidney disease, *GFR* glomerular filtration rate (GFR), *95 % CI* 95 % confidence intervals

### Secondary analyses

The staging of kidney function and the prevalence of any Albuminuria (≥30 mg/g) and/or eGFR (<60 ml/min/1.73 m^2^) at baseline based on Cockroft-Gault (CG) and CKD-EPI equations are shown in Table 2. Mean eGFR was lower based on CG and higher based on CKD-EPI, consistently in urban and rural areas, with significant sex differential in the distribution of eGFR across areas (both *p* ≤ 0.0003 for sex*residency interactions). The overall prevalence of decreased eGFR (<60 ml/min) was 21.9 % (95 % CI: 16.3-27.4) based on CG and 10.9 % (5.4-16.4) based on CKD-EPI, similarly in urban and rural areas (both *p* ≥ 0.242), with no evidence of sex*residence interactions (both *p* ≥ 0.09). The prevalence of the combined albuminuria and/or decreased eGFR was 35.8 % (27.3-44.2 %) based on CG, and 27.6 % (20.6-34.5) based on CKD-EPI. There was no rural vs. urban differences (both *p* ≥ 0.072); but there was evidence of significant interactions between gender and residence for both prevalence (both *p* ≤ 0.022 for sex*residence interaction), Tables 2.

## Discussion

This study revealed an overall high prevalence of CKD including albuminuria and stages G3-G4 as well as their related risk factors in this population, predominantly in the rural area, regardless the age and sex of participants. CKD and albuminuria were associated with history of hypertension and diabetes, elevated SBP and the presence of hypertension and diabetes, whereas existing diabetes and hypertension, and urban residency predicted the presence of stages G3-G4 CKD.

The high prevalence of CKD observed in this population has been reported in similar proportions in previous studies performed in SSA and low-to-middle income countries [8, 9, 19–21]. This result could be related to the higher prevalence and lower awareness of CKD and related risk factors observed in this population. Indeed, we found a high prevalence of CKD risk factors including diabetes, hypertension, and longstanding used of herbal and street medications which have been identified as predictors of CKD in this setting [9, 11, 20]. These results support the notion that Africans are at higher risk of CKD and confirm the high burden of CKD to be a worldwide phenomenon [3, 4, 22, 23]. However, variable CKD prevalence estimates have been published depending on the study design and setting, the target population characteristics, and the CKD definition and evaluation method [10–12, 24–26]. Compared to urban settings, CKD seems to be more prevalent in rural areas despite the lack of statistical difference as noticed in the metanalysis by Stanifer et al. [9]. The likely high prevalence observed in rural setting could be related to the high frequency of well-known clinical and socio-demographic risk factors for CKD occurrence and progression to ESRD [13].

In these apparently healthy populations, we observed that 2.5 % of the participants had CKD stages G3-G4. Numerous previous studies have reported a much higher prevalence of decreased eGFR (<60 ml/min/1.73 m^2^) based on a single assessment of the kidney function [8, 12, 19, 21, 24, 26, 27]. The difference would tend to confirm that studies based upon a single time-point assessment of kidney function overestimates the prevalence of CKD in population-based studies. However, some authors have also reported low prevalence of decreased eGFR based on single measurements, which could reflect true low prevalence, but also issues with the reliability of kidney function measurements including glomerular filtration rate estimation equations and the serum creatinine dosage method used [6, 10, 11].

Based on quantitative estimation of albuminuria, we observed a high prevalence of albuminuria with rural predominance and rates similar to those observed by Varma et al. in India using the same method [19]. Most published studies have reported much higher prevalence of albuminuria similar to those observed in our study population based only on the first measurement [7, 8, 12]. The semi-quantitative dipstick method of albuminuria assessment without confirmation test, the bias in reading the results and the presence of transient proteinuria could explain this high prevalence. Nevertheless, some authors have also reported low prevalence of albuminuria on dipstick with prevalence ranging from 2.25 to 7.4 %, which could reflect true lower risk of albuminuria in their population as well as bias in reading the results [10, 11, 25, 27].

The reported high prevalence of CKD, stages G3-G4 and albuminuria in our study, driven essentially by hypertension, diabetes, obesity and longstanding used of herbal and street medications invite policy makers to undertake an array of actions to promote and sustain nephroprotection. Such actions could include the implementation of an integrated CKD prevention and care programmes in order to promote the screening of high-risk populations, patient education and creation of multidisciplinary care teams. The implementation of such programmes has been shown to significantly reduce mortality, incidence of end stage renal disease and dialysis, and dialysis related cost [20].

### Strengths and limitations

The present study has some limitations including the non screening of participants for endemic infections conferring high risk of CKD such as HIV infection, hepatitis B and C viral infection; and the non-exhaustive assessment of socioeconomic status which has been shown to be associated with CKD [9, 21]. Moreover, by conducting this study in only one geographical site, despite its cosmopolitan population, there is little opportunity of assessing the variations in the prevalence of CKD across the country. However, this study to our knowledge is the first to provide community-based data on the epidemiology of kidney disease in the country using the optimal approach to CKD screening [13]. The inclusion of participants from a cosmopolite health district including an urban and rural area likely captures the diversity of the national population. Another major strength of this study is the provision of a complete picture of the CKD and albuminuria prevalence as well as their determinants in this population presenting a high prevalence of common risk factors of CKD.

## Conclusion

This study has revealed a high prevalence of CKD, stages G3-G4 and albuminuria in this setting, driven essentially by their commonest risk factors, and predominantly in rural area. These findings invite an array of actions to promote and sustain preventive measures to lower the risk of CKD, promote early detection of the disease and implementation of measures to slow the progression to the terminal stage of the disease.

## Additional file

Additional file 1: Table S1. The different clusters and corresponding health areas. (DOCX 15 kb)

## Abbreviations

BMI: Body mass index
CG: Cockroft-Gault
CKD: Chronic kidney disease
CKD-EPI: Chronic kidney disease epidemiology collaboration
DBP: Diastolic blood pressure
eGFR: Estimated glomerular filtration rate
MDRD: Modification of Diet in Renal Disease
SBP: Systolic blood pressure
SD: Standard deviation
