# Supplementary material for: Prevalence and determinants of chronic kidney disease in rural and urban Cameroonians: a cross-sectional study
Source: BMC Nephrol. 2015 Jul 30;16:117. doi: 10.1186/s12882-015-0111-8 (PMC4518633; doi:10.1186/s12882-015-0111-8)
Supplement: Additional file 1: Table S1. — The different clusters and corresponding health areas. (DOCX 15 kb) [file 12882_2015_111_MOESM1_ESM.docx]

**Supplemental Table 1: The different clusters and corresponding health areas.**

| **Health area** | **Population** | **Cumulative population** | **Cluster number** |
| --- | --- | --- | --- |
| Baleveng | 28214 | 28214 | G1 G2 G3 |
| Balevoni | 2535 | 30749 |  |
| Doumbouo | 22151 | 52900 | G4 G5 |
| **Fiala foreke (urban)** | 34391 | 87291 | G6 G7 G8 G9 |
| Fokoué | 10798 | 98089 | G10 |
| **Fometa (urban)** | 17707 | 115796 | G11 G12 |
| Fomopea | 3404 | 119200 |  |
| Fonakeukeu | 7210 | 126410 | G13 |
| Fondonera | 12942 | 139352 | G14 |
| Fongo ndeng | 10609 | 149961 | G15 |
| Fontsa toula | 5111 | 155072 |  |
| Fotetsa | 7179 | 162251 | G16 |
| Latchouet | 4300 | 166551 | G17 |
| Lepoh | 14302 | 180853 | G18 |
| Lingang foto | 8127 | 188980 | G19 |
| Maka | 14757 | 203737 | G20 |
| Mbeng | 28007 | 231744 | G21 G22 G23 |
| Mboua | 16980 | 248724 | G24 |
| Mekouale | 12383 | 261107 | G25 G26 |
| Ndoh jutsitsa | 18662 | 279769 | G27 G28 |
| Nkeuli | 7581 | 287350 |  |
| **Siteu (urban)** | 21935 | 309285 | G29 G30 |
| **Total** | **309285** | **309285** |  |
